# Impact of a Mindfulness Mobile Application on Weight Loss and Eating Behavior in People with Metabolic Syndrome: a Pilot Randomized Controlled Trial

**DOI:** 10.1007/s12529-023-10173-2

**Published:** 2023-03-21

**Authors:** Takaharu Matsuhisa, Rieko Fujie, Rie Masukawa, Natsue Nakamura, Norihisa Mori, Kazuyuki Ito, Yuki Yoshikawa, Kentaro Okazaki, Juichi Sato

**Affiliations:** 1 Department of General Medicine, Nagoya University Graduate School of Medicine, 65 Tsurumai-cho, Showa-ku, Nagoya, 466-8560 Japan; 2 Matsuhisa Clinic, 1-2-23 Shinmichi, Nishi-ku, Nagoya, 451-0043 Japan; 3 Fujita Health University School of Medicine, 1-98 Dengakugakubo, Kutsukake-cho, Toyoake, 470-1192 Japan; 4 Kasugai General Health Care Center, 1-1-1 Takaki-cho, Kasugai, 486-0804 Japan; 5 Community Medicine Education Unit, Faculty of Medical Sciences, Kyushu University, 3-1-1 Maidashi, Higashi-ku, Fukuoka, 812-8582 Japan; 6 Department of General Medicine, Nagoya University Hospital, 65 Tsurumai-cho, Showa-ku, Nagoya, 466-8560 Japan

**Keywords:** Mindfulness, Weight loss, Eating behavior, Metabolic syndrome, Mobile application

## Abstract

**Background:**

Weight-loss approaches involving mindfulness have been reported to reduce overeating behavior. We conducted a preliminary evaluation of the feasibility and effectiveness of a mindfulness mobile application (MMA) combined with a comprehensive lifestyle intervention (CLI) focused on weight loss and eating behaviors for people with metabolic syndrome based on post-intervention follow-up data.

**Method:**

Participants were randomly assigned (1:1) to a CLI group or a CLI + MMA group. Participants received weekly CLI for 13 weeks, followed by telephone counseling for 13 weeks. The CLI + MMA group also had access to the MMA. Feasibility was assessed by the number of people who refused to participate, rate of adherence to the MMA, follow-up rate, and participant satisfaction. The preliminary endpoint was weight change (at 26 weeks). Participants completed the Dutch Eating Behavior Questionnaire (DEBQ). A mixed linear model was used for efficacy analysis.

**Results:**

Eight of the 40 participants declined to participate. The MMA was used 4.4 ± 1.7 days per week, but the rate of adherence declined over time. The follow-up rate was 100%, and there was no difference in participant satisfaction between the groups. There was no significant group-by-time interaction for weight loss (*p* = 0.924), but there was a significant interaction for the DEBQ restrained eating score (*p* = 0.033).

**Conclusions:**

This study found that CLI plus MMA was highly feasible and moderately acceptable. There were no significant differences in weight loss between the groups, but the CLI + MMA group showed an increase in restrained eating. Further large-scale studies are needed.

**Trial Registration:**

Japanese University Hospital Medical Information Network (UMIN-ICDR). Clinical Trial identifier number UMIN000042626.

## Introduction

Increasing obesity rates are an international problem [1], including in Japan [2]. Obesity is a major cause of metabolic syndrome (MetS) [3]. Reducing obesity and improving MetS can improve hypertension, glucose intolerance, and dyslipidemia, and reduce the morbidity and mortality associated with cardiovascular diseases [4]. Aggressive lifestyle modification focused on weight loss and increased physical activity is the principal treatment for improving MetS [5]. Although intensive lifestyle interventions involving diet and exercise therapy are effective for people with obesity in the short term, maintaining long-term weight loss is difficult [6–8]. Different dysfunctional eating behaviors, such as binge eating, emotional eating, external eating, and eating in response to food cravings, have been linked to weight regain after successful weight loss [9]. This highlights the importance of investigating approaches to address associated psychological problems and potentially increase motivation and self-control among patients with obesity (e.g., to limit impulsive and inappropriate use of food) [10]. Psychological interventions, particularly behavioral and cognitive-behavioral strategies, have been reported to be beneficial for weight loss among adults with overweight and obesity, especially when combined with dietary and exercise strategies [11].

Mindfulness is a psychological process in which attention is paid to experiences that occur in the present moment. When developed through meditation and other disciplines, mindfulness can improve emotional control and reduce avoidant reactions to external and internal experiences [12]. Mindfulness-based interventions have been shown to have beneficial effects on weight loss and impaired eating behaviors, including improving the present-moment awareness of the sensory properties of food and reducing further food intake, and supporting decentering strategies that may help individuals resist desired foods [13]. Previous research suggested that mindfulness positively affected weight-related behaviors, such as reducing emotional and binge eating [9, 14–19], but had mixed effects on weight loss [14, 16–18, 20]. However, few studies have focused on evaluation at follow-up after an active intervention. A systematic review and meta-analysis found that a weight-loss approach involving mindfulness reduced overeating behavior and contributed to maintaining weight loss at follow-up [14]. A small-scale randomized controlled trial (RCT) showed that mindfulness combined with a standard behavioral weight loss program resulted in better weight loss, less overeating behavior, and better adherence to dietary restrictions at the 3-month follow-up after a 3-month active intervention compared with a standard behavioral weight loss program [21]. These studies suggested that mindfulness may be effective for maintaining long-term weight loss and healthy eating behavior after an active intervention.

Mindful eating (derived from mindfulness), when used to address unhealthy eating behavior, incorporates nonjudgmental awareness of physical and emotional sensations associated with eating [22]. Although research on mindful eating has been used in various ways, experimental studies are relatively limited, and it is not yet possible to conclude whether mindful eating strategies impact diet. However, some evidence suggests that certain mindful eating strategies may be promising, such as decentering and attention to the sensory properties of food [23]. A previous review showed that a mindful eating intervention reduced cravings and excess caloric intake and helped maintain continued weight loss [24].

Some studies have reported non-face-to-face weight loss interventions using mindfulness [25, 26]. One such study found that a telephone intervention using a mindfulness-based weight loss program did not improve weight loss compared with a standard weight loss program at the end of the intervention period and at the 6-month follow-up [25]. However, participants who used mindfulness in conjunction with the intervention showed decreased overeating behavior and improved mindful eating practices and mindfulness scale scores [25]. To date, the only published intervention study involving a mindfulness mobile application (MMA) for weight loss involved a general sample of students and reported a comparison with a behavioral self-monitoring electronic diary (e-diary) method [26]. Those results revealed no weight loss in the MMA group at follow-up, although improvements were observed in participants’ stress, eating behavior, mindfulness, and the frequency of mindful eating practices [26]. However, no intervention studies have examined changes in weight and eating behaviors when an MMA is used in addition to diet and exercise therapy. Mobile application-based interventions have been proposed as useful tools for weight loss [27, 28]. These interventions are portable and easy to practice anywhere [12], which may contribute to their potential role in MetS care [29]. Therefore, we aimed to explore the potential effectiveness and feasibility of using an MMA in conjunction with a comprehensive lifestyle intervention (CLI) focused on weight loss and eating behaviors, including at the post-intervention follow-up.

## Methods

### Study Design

This open-label, parallel, pilot RCT included a 13-week CLI, which comprised a supportive workshop after a physical examination at a city general health center plus telephone counseling every 4 weeks for the following 13 weeks with and without MMA use. The application was provided to all participants, but the provision period was divided into two phases: 1–26 weeks and 27–52 weeks. We compared data between the two groups over 26 weeks. Participants were stratified by sex and randomly assigned to the two groups using a 1:1 ratio. After allocation, participants underwent baseline assessment, and those who did not meet the eligibility criteria were excluded from the analyses. The intervention was provided free of charge. This pilot trial was not designed to have sufficient statistical power to assess the effectiveness of the MMA intervention on weight loss and eating behavior.

### Participants

Eligibility criteria were used to select adults aged 20–75 years with MetS, as defined by the International Diabetes Federation (IDF) [30] that had smartphones with mobile applications available. The exclusion criteria were a history of serious heart disease or other conditions that prohibited exercise therapy, severe depression, severe anxiety disorder, severe somatoform disorder, and psychotic symptoms. We recruited 1031 participants (663 females) by mail who met the eligibility criteria from among individuals who underwent physical examinations at a general healthcare center in Kasugai City, Japan. Participants were recruited from April 2020 to January 2021. Three evaluation sessions (baseline, week 13, and week 26) were conducted at the general healthcare center.

### Randomization, Blinding, and Allocation Concealment

After orientation, participants who consented to participate in this study were randomly assigned to one of the two groups using the envelope method stratified by sex. The envelopes were opened in sequence in front of the participants. Because this was a pragmatic comparative study rather than a placebo-controlled trial, it was not possible to blind participants or the health workers providing the CLI. However, the risk for detection bias was minimized because all sessions were conducted independently, and the nutritionists and public health nurses in charge of the CLI were not involved in evaluating the results. The statistician always conducted outcome assessments in a separate room and was blinded to group allocation.

### Interventions: CLI

The CLI involved a 1-h lesson once a week for 13 weeks from April to June 2021 and was performed with all participants together at designated times. The sessions comprised lectures by public health nurses and nutritionists, nutritional guidance, and lectures and exercise guidance by health exercise instructors. Nutritional guidance was based on the portion control method using the Healthy Plate approach [31], with a daily diet of 1200–1500 kcal plus dairy products and fruits, with a target of 50% carbohydrate, 25–30% protein, and 20–25% fat. The exercise instruction included open-eyed one-legged stands, heel lifts, squats, arm-leg crossing, push-ups, and sit-ups, with an increased load each time. Participants recorded their weight, exercise, meals, and snacks in a notebook each day. They were interviewed and given written feedback on their notebooks by public health nurses and nutritionists when they attended the sessions. As a state of emergency in response to COVID-19 was declared in Aichi Prefecture from May 12 to June 20, 2021 [32], six of the sessions scheduled during this time were canceled. Therefore, only six sessions were held. During the state of emergency, nutritionists and public health nurses called participants once a week to check on their condition. After completion of the active intervention, the nutritionists and public health nurses conducted telephone counseling every 4 weeks from July to September (weeks 14–26) to check whether participants were able to maintain the diet and exercise program and confirm that the MMA group was using the application. These healthcare workers provided instruction to participants based on the transtheoretical model of health behavior change [33].

### Experimental Intervention: CLI + MMA

The CLI + MMA group performed the CLI and practiced mindfulness every day using the mobile application. Participants’ MMA use was checked and counseling provided by the nutritionists and public health nurses (who were not experienced in mindfulness practices) when they delivered sessions or telephone counseling. We used the MMA developed by the Relook unit of ARETECO HOLDINGS LTD (https://relook.jp/) for this study, which comprised a 26-week MMA program for weight loss. The present researchers were not involved in the creation of this program. The application was provided free of charge and participants downloaded the application onto their smartphones. Details of the mobile application content are shown in Table 1. The CLI + MMA intervention comprised an average of 453 s (306–889 s) each day, and the application included a reminder notice once-a-day. Participants initially practiced basic mindfulness breathing exercises following audio navigation in Japanese using the mobile application. After practicing mindfulness breathing, participants practiced ways to be mindful in various daily situations. The MMA was provided free of charge to the CLI group for 26 weeks after the evaluation was completed to ensure equality between the two groups.

**Table 1:** Content of the mindfulness mobile application

Session	Theme	Session purpose	Content of the session
1st (weeks 1–6)	Learn about and experience mindfulness	To grasp a sense of mindfulness through both intellectual knowledge of mindfulness and experience To develop a habit of daily mindfulness meditation To learn how to prepare for mindfulness	Mindful eating (7 days), 3-min breathing exercise (3 days), 5-min breathing exercise (14 days), silent contemplation (14 days), body scan meditation (2 days), SOBER breathing space (2 days)
2nd (weeks 7–12)	Increase attention and awareness	To become aware of thoughts, judgments, emotions, and feelings To pay attention more easily than in weeks 1–6	Mindful eating (5 days), body scan meditation (5 days), walking meditation (7 days), silent contemplation (14 days), RAIN (6 days), SOBER breathing space (5 days)
3rd (weeks 13–18)	Practice mindfulness on a daily basis	To implement mindful coping strategies for stress To expand the attention beyond breath and body, including sound, thought, and being	Mindful eating (6 days), self-soothing touch (7 days), self-compassion break (14 days), RAIN (8 days), silent contemplation (7 days)
4th (weeks 19–26)	Review mindfulness	To memorize the mindfulness mindset To know how to perform mindfulness To understand the importance of continued mindfulness	Mindful eating (2 days), 3-min breathing exercise (4 days), 5-min breathing exercise (11 days), RAIN (3 days), SOBER breathing space (3 days), silent contemplation (11 days), body scan meditation (9 days), self-soothing touch (3 days), self-compassion break (5 days), walking meditation (5 days)

*SOBER* Stop Observe Breathe Expand, and Respond, *RAIN* Recognize, Allow, Investigate, and Nurture

### Outcomes

#### Acceptability, Adherence, and Feasibility

These outcomes were measured by the number of people who refused to consent to the study after receiving orientation, the rate of adherence to the application (number of days application implementation was completed), follow-up rate, participant satisfaction, and number of adverse events. Data on patient adherence to the application were collected by ARETECO HOLDINGS LTD, which was performed automatically by the app and recorded every time a participant completed more than 90% of an application session. ARETECO HOLDINGS LTD provided the research team with a list of codes, which allowed the researchers to see a log of the actual time the application was used. Satisfaction was assessed at week 26, with reference to previous studies [25], to compare the satisfaction of participants in the two groups regarding their overall impression of the program using a 5-point Likert scale (1 = “very dissatisfied” to 5 = “very satisfied”). We also assessed participants’ willingness to recommend the program to friends, and whether the program helped them consume a healthy diet using a 5-point Likert scale (1 = “very negative” to 5 = “very positive”). We systematically tracked adverse events at each weekly session and followed monthly telephone counseling.

### Assessment procedures

The primary, secondary, and exploratory outcomes were evaluated at weeks 0, 13, and 26.

#### Primary Outcome Measures

The primary outcome of using the MMA was the rate of change in body weight. Body weight was measured in units of 0.1 kg, while wearing test clothes.

#### Secondary Outcome Measures

Eating behavior.

The modified Japanese version of the Dutch Eating Behavior Questionnaire (DEBQ) was used to evaluate eating behavior. The DEBQ has three eating behavior subscales: restrained eating, emotional eating, and external eating [34]. The Japanese version of the DEBQ has been reviewed for reliability [35]. The 10-item restrained eating subscale assesses intentions and behaviors regarding restricting food intake because of weight concerns. The 13-item emotional eating subscale rates overeating behaviors triggered by negative emotions, such as anger, boredom, anxiety, and fear. The 10-item external eating subscale measures eating in response to food-related stimuli, such as the smell and taste of food, seeing other people eating, and seeing food being prepared. Participants responded to each item on a 5-point scale from “never” (1 point) to “always” (5 points). Higher scores indicated greater endorsement of that eating behavior.

#### Exploratory Outcome Measures

As exploratory outcome measures, we assessed body mass index (BMI), body fat percentage, abdominal circumference, body blood pressure, and blood test parameters (total cholesterol, high-density lipoprotein, low-density lipoprotein, triglyceride, fasting blood sugar, hemoglobin A1c, blood urea nitrogen, creatinine, cystatin C, estimated glomerular filtration rate), self-reported physical activity using the Japanese version of the International Physical Activity Questionnaire (short version) [36], and the Motivation to Live a Healthy Diet scale [37]. Because of the large amount of data collected, this paper only describes the results for body weight and eating behavior, as no association between the other items and the effects of mindfulness was shown.

### Statistical Analysis

Statistical analyses were performed using SPSS version 27.0 (IBM Corporation, Armonk, NY, USA). After group allocation and before the intervention, one participant refused to participate in this study. Another participant was excluded because they did not meet the IDF criteria for MetS at the baseline assessment. Therefore, the modified intention-to-treat method was applied to investigate the treatment effects. Continuous variables measured at baseline were described using means and standard deviations and compared between treatment groups using two-sample *t*-tests. Categorical variables were described using frequencies and percentages, and comparisons between treatment groups were made using Fisher’s exact tests (two-sided). We used linear mixed models to analyze the effects of the intervention on body composition and DEBQ scores, including the endpoint of weight change. The model included fixed effects of group, time, and group-by-time interactions. The linear modeling analysis of participants who completed the MMA on at least 5 out of 7 days (per protocol) supported the results and the conclusions of the linear mixed modeling for the full sample. In this study, we only report the results of the linear mixed modeling for the full sample. It is considered more appropriate to use the standard deviation of the baseline values to reflect clinically meaningful differences based on the distribution of the population [38]. The effect size was calculated as the difference in mean changes at week 26 between the two groups, standardized to the pooled standard deviation of the baseline values. As an additional analysis, the rate of weight loss, changes in DEBQ score, and the frequency of application use were analyzed using Pearson’s product-moment correlation coefficients.

## Results

### Participant Flow: Screening and Study Acceptability

**Fig. 1:**
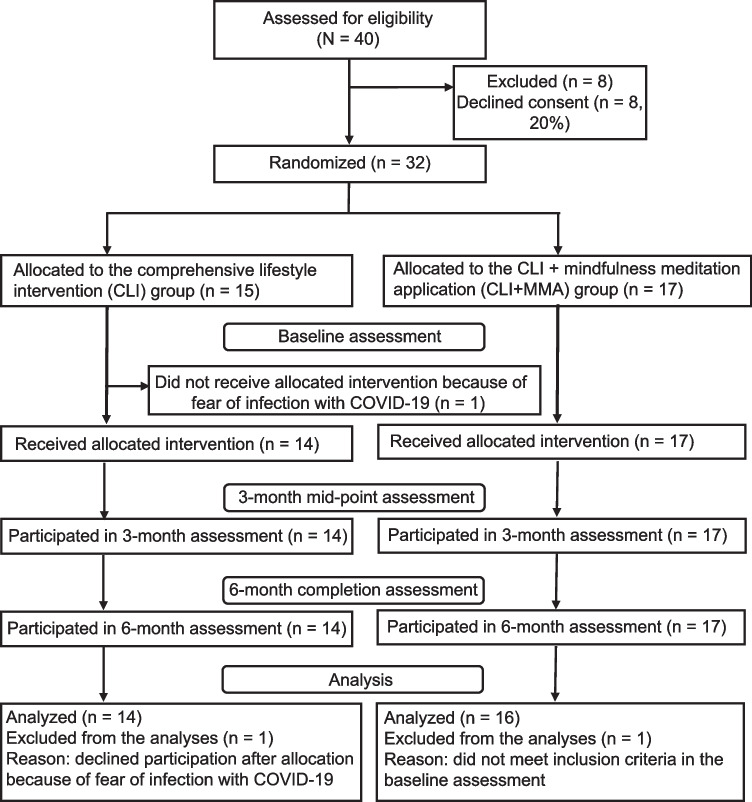
Flow diagram of trial participants

In total, 40 people attended the study orientation, of which 8 (20%) declined to participate in this study. The remaining 32 participants (80%) were randomly assigned to the study groups: 17 to the CLI + MMA group and 15 to the CLI group. One participant refused to participate in this study because of fear of infection with COVID-19 before the intervention started. Another participant did not meet the inclusion criteria for baseline measurements. These two participants were therefore excluded from the analyses, and background characteristics and results are reported for 30 participants. The enrollment, randomization, and retention processes are shown in Fig. 1.

### Participants’ Characteristics

Differences between the two groups at baseline are shown in Table 2. Most participants (83%, *n* = 25) were female and the mean age was 69.3 ± 5.0 years. Baseline measurements for the two groups showed a significant difference in weight (*p* = 0.035), with mean weights of 61.7 ± 7.5 kg and 69.3 ± 11.0 kg in the CLI + MMA and CLI groups, respectively.

**Table 2:** Baseline characteristics of participants randomly assigned to the comprehensive lifestyle intervention (*CLI*) and the CLI plus mindfulness mobile application group, assuming intention-to-treat

	CLI + mindfulness (*n* = 16)	CLI (*n* = 14)	*p*-value* (two-tailed)
Demographics			
Gender, female	13 (81.3)	12 (85.7)	1.000
Age (years)	69.9 ± 3.6	68.7 ± 6.3	0.535
**Baseline measures**			
Height, cm	154.9 ± 5.3	158.1 ± 7.5	0.182
Weight, kg	61.7 ± 7.5	69.3 ± 11.0	0.035
Body mass index, kg/m^2^	25.7 ± 2.2	27.6 ± 3.2	0.055
Abdominal circumference, cm	93.6 ± 6.5	99.0 ± 9.0	0.065
Body fat percentage, %	34.3 ± 4.4	38.2 ± 6.7	0.069
Systolic blood pressure, mmHg	135.9 ± 17.5	133.0 ± 11.9	0.608
Diastolic blood pressure, mmHg	76.2 ± 11.1	77.6 ± 11.4	0.739
Total cholesterol, mg/dL	211.9 ± 45.9	208.6 ± 33.7	0.826
LDL cholesterol, mg/dL	123.4 ± 36.8	121.5 ± 32.8	0.881
HDL cholesterol, mg/dL	68.0 ± 15.1	62.6 ± 13.0	0.305
Triglyceride, mg/dL	171.4 ± 62.4	195.4 ± 94.9	0.413
Blood sugar, mg/dL	115.8 ± 23.3	130.9 ± 53.9	0.316
Hemoglobin A1c, %	6.0 ± 0.5	6.6 ± 1.2	0.107
Blood urea nitrogen, mg/dL	17.5 ± 3.8	18.6 ± 3.6	0.393
Creatinine, mg/dL	0.71 ± 0.11	0.72 ± 0.25	0.868
Cystatin C, mg/L	0.85 ± 0.14	0.84 ± 0.16	0.835
eGFR, mL/min/1.73 m^2^	66.9 ± 8.8	68.9 ± 15.8	0.678
Restrained eating (DEBQ)	3.27 ± 0.69	3.54 ± 0.53	0.251
Emotional eating (DEBQ)	1.95 ± 0.75	2.10 ± 0.84	0.614
External eating (DEBQ)	2.97 ± 0.73	2.84 ± 0.54	0.581
Total physical activity, MET-minutes/week	2319.6 ± 3017.8	1487.2 ± 1402.5	0.353
Sedentary time, minutes	384.4 ± 223.2	345.0 ± 216.6	0.629
Presence of chronic diseases			
None	6 (37.5)	4 (28.6)	0.709
All	10 (62.5)	10 (71.4)	
The number of times the course was taken			
First	10 (62.5)	6 (42.9)	0.464
Plural	6 (37.5)	8 (57.1)	

Data are presented as mean ± standard deviation or n (%). *LDL*, low-density lipoprotein; *HDL*, high-density lipoprotein; *eGFR*, estimated glomerular filtration rate; *DEBQ*, Dutch Eating Behavior Questionnaire; *MET*, metabolic equivalent

^*^Group comparisons by two-sample *t*-tests for continuous data and Fisher’s exact test (two-sided) for categorical data

### Acceptability, Adherence, and Feasibility

**Fig. 2:**
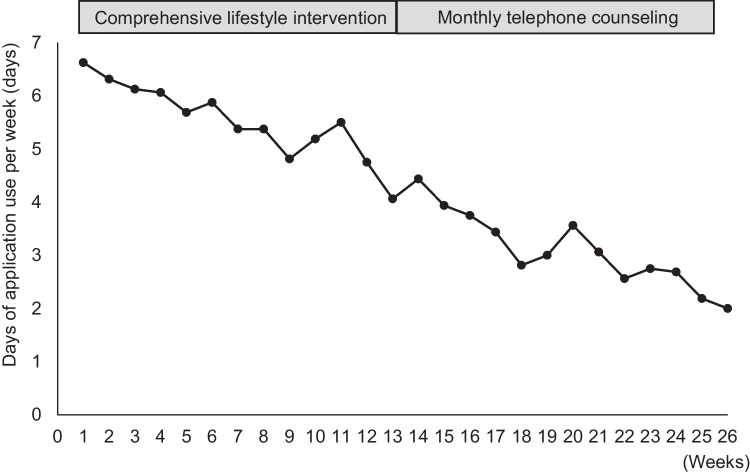
Average number of days the application was used per week. The average number of days spent using the application per week declined with time

After 6 months of evaluation, data were available for all 30 participants (follow-up rate: 100%). No adverse events were observed. The application use logs revealed the application was used 4.4 ± 1.7 days per week on average; however, the number of days of application use decreased over time (Fig. 2). In the first week, the average number of days of application use was 6.63 ± 0.78, which decreased to 4.06 ± 2.56 in week 13, and 2.00 ± 2.34 in week 26. The number of participants who did not use the MMA by intervention week was 0 at week 1, but increased to two of 16 (17.5%) at week 13, and eight of 16 (50%) at week 26. Sixteen CLI + MMA participants (response rate = 100%) and 14 CLI participants (response rate = 100%) reported satisfaction with their participation in the 6-month survey. The CLI + MMA and CLI groups had similar mean program satisfaction ratings for overall impressions of the program (CLI + MMA: 3.4 ± 0.9; CLI: 3.5 ± 1.2), recommending the program to friends who wanted to lose weight (CLI + MMA: 3.4 ± 1.0; CLI: 3.5 ± 1.0), and helping them to eat healthily (CLI + MMA: 3.8 ± 0.7; CLI: 3.4 ± 0.9).

### Weight Loss

**Fig. 3:**
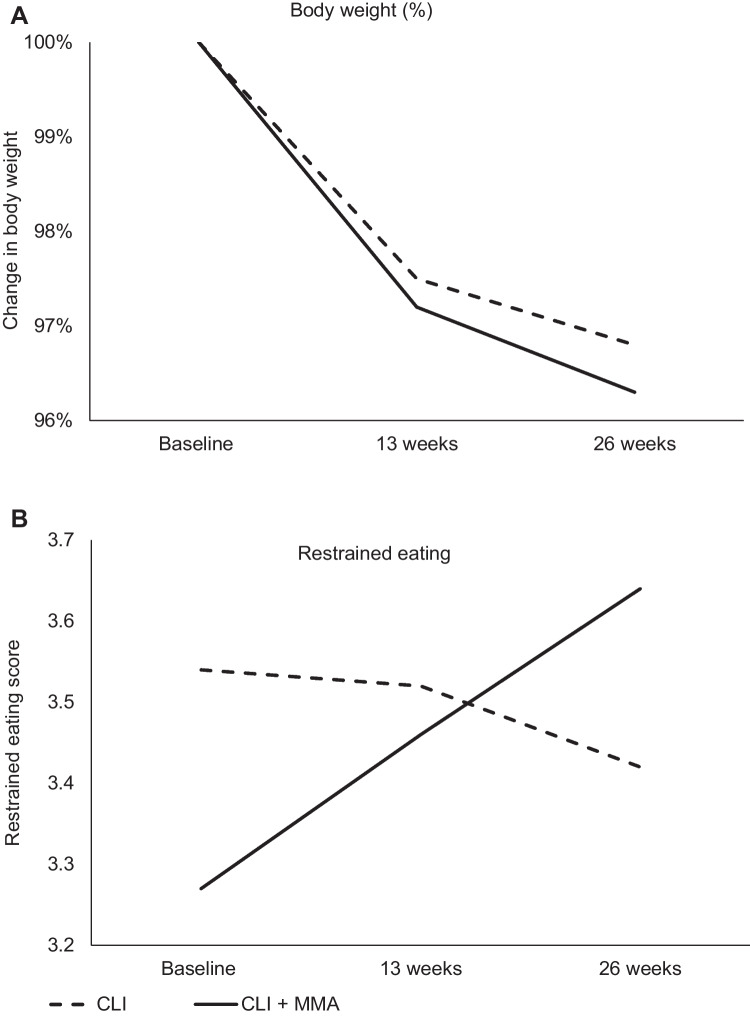
Change in body weight (%) (**A**) and restrained eating score (DEBQ) (**B**) for CLI versus CLI + MMA

Compared with baseline, the mean weight loss at week 26 was significantly reduced by 2.1 kg (standard error (SE) 0.6, *p* = 0.004; 3.2%, SE 0.9) in the CLI group and 2.3 kg (SE 0.2,* p* < 0.001; 3.7%, SE 0.4) in the CLI + MMA group. There was no significant group-by-time interaction between mindfulness use and weight loss (*p* = 0.924). These results are shown in Table 3 and Fig. 3.

**Table 3:** Differences in primary and secondary outcomes (intention-to-treat) by group over time based on linear mixed modeling (CLI: *n* = 14; CLI + MMA: *n* = 16)

Outcome variable	By intervention group	Assessments			*p*-values			Effect size
		Week 0	Week 13	Week 26	Group effect	Time effect	Group-by-time interaction	Baseline to 6 months
		Mean (standard error)						
Primary outcome								
Body weight, kg	CLI	69.3 (2.9)	67.6 (3.0)	67.2 (3.2)	0.039	< 0.001	0.924	0.02
	CLI + MMA	61.7 (1.9)	60.1 (2.0)	59.5 (2.0)				
Secondary outcomes								
DEBQ Restrained eating	CLI	3.54 (0.14)	3.52 (0.15)	3.42 (0.16)	0.850	0.354	0.033	0.78
	CLI + MMA	3.27 (0.17)	3.46 (0.13)	3.64 (0.16)				
Emotional eating	CLI	2.10 (0.22)	1.95 (0.17)	2.02 (0.25)	0.629	0.412	0.419	0.10
	CLI + MMA	1.96 (0.19)	1.98 (0.14)	1.79 (0.15)				
External eating	CLI	2.84 (0.15)	2.72 (0.16)	2.87 (0.18)	0.930	0.188	0.269	0.51
	CLI + MMA	2.97 (0.18)	2.73 (0.15)	2.68 (0.16)				

*DEBQ* Dutch Eating Behavior Questionnaire, *CLI* comprehensive lifestyle intervention, *MMA* mindfulness mobile application

### DEBQ

Analysis of DEBQ results showed the restrained eating score significantly increased from 3.27 (SE 0.17) to 3.64 (SE 0.16) at week 26 in the CLI + MMA group (*p* = 0.010), but no change was observed in the CLI group (baseline: 3.54, SE 0.14; week 26: 3.42, SE 0.16). The group-by-time interaction was significant (*p* = 0.033) (Fig. 3B and Table 3). The DEBQ emotional eating score showed a decreasing trend in the CLI + MMA group from 1.96 (SE 0.19) to 1.79 (SE 0.15) at week 26, but the difference was not significant (*p* = 0.311). There was no change in emotional eating score in the CLI group (baseline: 2.10, SE 0.22; week 26: 2.02, SE 0.25) and no significant group-by-time interaction (*p* = 0.419). The external eating score showed a decreasing trend in the CLI + MMA group from 2.97 (SE 0.18) to 2.68 (SE 0.16) at week 26, but this difference was not significant (*p* = 0.098). No changes were observed in the CLI group (baseline: 2.84, SE 0.15; week 26: 2.87, SE 0.18) and the group-by-time interaction was not significant (*p* = 0.269) (Table 3).

### Mechanisms/Moderators

**Fig. 4:**
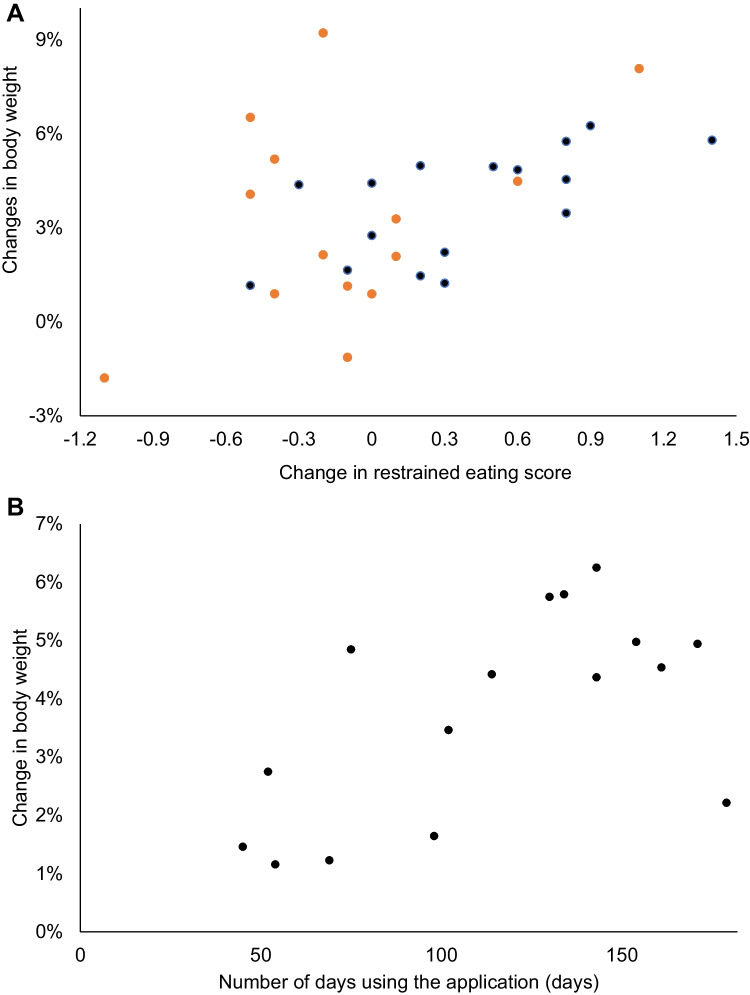
Correlation with weight loss: change in restrained eating score (DEBQ) (**A**) and application use days (**B**). **A** The CLI + MMA group: black dots; the CLI group: orange dots

We examined potential mechanisms for mindfulness treatment by correlating the change between baseline and follow-up at week 26 (follow-up score minus baseline score) for the rate of weight loss and restrained eating scores (Fig. 4A). Overall, an increase in restrained eating scores was correlated with higher rates of weight loss (Pearson’s correlation coefficient: 0.474; Fig. 4A), especially in the CLI + MMA group (Pearson’s correlation coefficient: 0.634; Fig. 4A, black dots) compared with the CLI group (Pearson’s correlation coefficient: 0.417; Fig. 4A, orange dots). We also investigated mindfulness engagement to test whether more engagement with the MMA was associated with more weight loss. In the CLI + MMA group, the number of days spent using the MMA was significantly and positively associated with increased weight loss (Pearson’s correlation coefficient: 0.598, *p* = 0.014; Fig. 4B).

## Discussion

The present study showed the combination of CLI and MMA did not affect participants’ satisfaction with the program over 26 weeks. The high follow-up rate in this study allowed us to successfully track participants, but the rate of adherence to the MMA was low. No adverse events were observed. The preliminary results indicated that the MMA intervention did not result in greater weight loss at 6 months, but significantly increased restrained eating behavior compared with the CLI group. A positive relationship was found between the rate of weight loss and increased restrained eating scores. There was also a positive relationship between the rate of weight loss and frequency of using MMA. These results suggested the MMA was potentially effective for people with MetS.

Weight loss results did not significantly differ between the CLI + MMA and CLI groups at follow-up from the end of the CLI. Overall, both groups exhibited modest weight loss (3.5% of body weight, 2.2 kg on average). These effects on body weight were consistent with the results of previous meta-analyses, which reported small effect sizes for weight loss with mindfulness-based interventions [17–19]. A meta-analysis demonstrated moderate effects on weight loss with these interventions, but the level of evidence was limited because of methodological weaknesses and variability [20]. The present study was designed with the CLI group as the target group, and the results were similar to those of the most recent meta-analysis in that the interventions in both groups resulted in the same amount of weight loss [17]. Regarding the persistence of intervention effects on weight loss, weight regain after an active intervention is common, with around half of the lost weight being regained within 2 years [39]. Carriere et al. conducted a systematic review and meta-analysis that revealed a difference in weight at follow-up with and without the intervention [14]. However, there is variability in the timing of weight-related assessments among studies [40], and it is unclear when weight regain occurs. A small-scale RCT examining a mindfulness intervention reported weight gain in the CLI group after 12 weeks of follow-up [21]. However, in the present study, both the CLI + MMA and CLI groups exhibited no weight gain. This discrepancy could be attributed to differences in study methodology, as well as differences in the average age and ethnicity of the sample. In this study, we continued regular telephone-based counseling after the active intervention. Continued biweekly or monthly behavioral counseling after initial weight loss is known to be an effective approach for preventing weight regain [41]. Therefore, the follow-up period in our study might have been insufficient to assess the impact of the MMA itself on weight, meaning the results do not preclude the possibility that the MMA intervention may help weight loss. Further large-scale, long-term follow-up studies are needed to confirm our findings.

Regarding eating behavior, we found restrained eating behavior was significantly increased in the CLI + MMA group. Several meta-analyses reported that mindfulness improved binge eating behavior [9, 14–19]. Daubenmier et al. measured the effect of mindfulness using the DEBQ as an evaluation item [42], and revealed a small effect on restrained eating behavior, but large effects on emotional eating and external eating behavior [42]. In the present study, the effects on restrained and external eating behavior were large, and the effects on emotional eating behavior were small. Compared with the previous study, participants in our study reported healthier eating behaviors in their baseline assessment (our study: restrained eating 3.27, emotional eating 1.95, external eating 2.97; Daubenmier et al. [42]: restrained eating 2.79, emotional eating 3.42, external eating 3.57). In addition, participants in the present study were older and had a lower BMI than those in the previous study [42]. Further research is needed to elucidate this issue in more depth.

The MMA used in this study appeared to be feasible, as most participants with MetS found it acceptable and the loss to follow-up was small. However, the average adherence to the application declined over time. The overall average length of time the MMA was used each day was 453 s (range 306–889 s) (Fig. 2). Maintaining adherence is important in weight management [27], and our sensitivity analysis showed a correlation between adherence to the MMA and weight loss (Fig. 4B). A systematic review and meta-analysis of home practice in mindfulness-based cognitive therapy and mindfulness-based stress reduction showed the pooled estimate for participants’ home practice was 64% of the assigned amount, equating to about 30 min/day, 6 days/week [43]. Similarly, a systematic review of cancer survivors’ adherence to home mindfulness practice found the pooled adherence rate for participants’ home practice was 60% of the assigned amount (27 min/day during the intervention period), although survivors tended to practice less as time passed [44]. A possible reason for this difference in adherence is that adherence to an application partly depends on users’ characteristics [45]. Participants in our study may not have been aware of the importance of mindfulness. In addition, the healthcare workers who checked MMA adherence were not familiar with mindfulness, which might have made it difficult to follow the transtheoretical model of health behavior change [33]. This study also included participants that were older than expected. Application-based studies with older participants have used devices with easy-to-use touch screens of about 7 in. [46] or incorporating multiple alarm functions per day [46, 47]. Creative strategies to increase adherence to such applications should be considered in further studies to comprehensively evaluate the benefit of the MMA for individuals with MetS.

The strength of the present study was our finding that increased restrained eating behavior may be associated with weight loss during CLI. Restraint theory suggests that cognitive control of human eating behavior leads to reduced sensitivity to internal cues for satiety, which can result in disinhibited eating (i.e., overeating) in situations where cognitive control is undermined [48]. However, there is little experimental evidence from non-clinical samples that increased eating restraint is related to disinhibited eating or an increased cognitive bias for food [49]. When those who reported trying to lose weight via dieting were compared with those who were not dieting, the former group showed a reduced frequency of binge eating [50]. Another study found current ongoing dieters did not show activation in the prefrontal cortex and orbitofrontal cortex regions related to cognitive control [51]. It is possible that participants in the present study had lost weight because they were ongoing dieters during CLI. In this study, the correlation between weight and restrained eating was higher in the CLI + MMA group compared with the CLI group (Fig. 4A). Mindfulness decreases default mode network and frontoparietal network connectivity, and changes in medial prefrontal-amygdala connectivity are critically implicated in the regulation of emotion, which may be related to improved emotional regulation [52, 53]. Although there is a possibility of confounding bias from other factors that contribute to weight loss, our findings regarding the MMA warrant further research.

The present study also had several limitations that should be considered. As expected in a pilot feasibility study, the sample size was too small to draw definitive conclusions. Furthermore, 92% of the sample were women, which limited the generalizability of the findings to men. A third limitation was the choice of a control intervention. We compared a CLI group as the control group because of the feasibility of the study. Although this was a proprietary program in the public interest and not a scientifically proven effective program for weight loss, similar interventions throughout Japan have shown some effectiveness [54]. Fourth, the randomization was not successful as the control group was significantly heavier than the intervention group at baseline. Fifth, this study was conducted during the COVID-19 pandemic [32]. In addition to the fact that the planned interventions could not be implemented, the restrictions on daily life caused by COVID-19 measures also affected eating and physical activity [55], which could have influenced the outcomes in our study.

The present study showed that the MMA was highly feasible and moderately acceptable for use by people with MetS. The average duration of MMA use in the present study was 453 s (range 306–889 s) per day. This was a short duration compared with traditional mindfulness interventions, which typically involve face-to-face group exercises lasting 2–2.5 h per session [56]. We found the effects of the MMA included improved eating behavior, which suggested that this low-intensity application was effective for modifying some behaviors and attitudes related to problem eating behavior. Further large-scale studies are needed to confirm our results. We also found an association between adherence to the MMA and weight loss. This could be attributable to the characteristics of the application or the relationship between participants and the healthcare workers delivering the program. However, it was difficult to fully examine this important factor using the data collected in this study. Further studies focused on this aspect may inform development of more effective interventions.

## Conclusion

This study is the first pilot RCT to examine the efficacy of MMA combined with diet and exercise therapy for MetS. This pilot study found that MMA with CLI was highly feasible and moderately acceptable, but low adherence to the application suggests that the intervention should include additional steps to improve engagement with the application. We found no difference in the effects on weight loss between the study groups, although use of the MMA might have contributed to an increase in restrained eating. Further large-scale studies building on this study are needed to demonstrate the effectiveness of MMA interventions.

## Data Availability

The datasets generated and analyzed during the current study are not publicly available due to confidentiality but are available from the corresponding author on reasonable request.
